# Prevalence of sensitive teeth and associated factors: a multicentre, cross-sectional questionnaire survey in France

**DOI:** 10.1186/s12903-020-01216-1

**Published:** 2020-08-26

**Authors:** Alessandra Blaizot, Damien Offner, Gilda Trohel, Valérie Bertaud, Christophe Bou, Céline Catteau, Camille Inquimbert, Laurence Lupi-Pegurier, Anne-Marie Musset, Paul Tramini, Jean-Noel Vergnes

**Affiliations:** 1 Dental Public Health Department, University of Lille, Faculty of Dentistry, Lille, France; 2 Collège National des chirurgiens-dentistes universitaires en santé publique (CNCDUSP) -French Society for Dental Public Health, Toulouse, France; 3 Faculty of Odontology, Dental Public Health Department, University of Strasbourg, Strasbourg, France; 4 University of Rennes, Faculty of Dentistry, Rennes, France; 5 University of Bordeaux, Faculty of Odontology, Bordeaux, France; 6 Faculty of Odontology, Dental Public Health Department, University of Montpellier, Montpellier, France; 7 University of Côte d’Azur, MICORALIS, EA 7354 Nice, France; 8 Dental Public Health Department, Paul Sabatier University, Toulouse, France

**Keywords:** Oral health, Dentin hypersensitivity, Observational study, Questionnaire, Prevalence, Logistic model

## Abstract

**Background:**

As far as we know, little data, whether obtained from self-administered questionnaires or upon dental clinical examination, has been published on the prevalence of sensitive teeth (ST) in the French adult population. The objectives of the present work were to estimate ST prevalence and characteristics in the general population of France and to explore the associated factors.

**Method:**

A multicentre cross-sectional study was conducted between November 2011 and March 2013 in six French cities. Adult passers-by in public places were invited to answer an electronic questionnaire on a tablet computer. Only people who declared having at least one natural tooth were included in the study. A logistic regression model was used for the multivariate analysis.

**Results:**

The prevalence of ST during the previous 12 months reported by the sample of 2413 participants was 42.2% [95% CI: 40.2–44.1%]. The final logistic regression model showed significant statistical associations between ST and female gender, use of tobacco, consumption of soft drinks, limited access to oral care and poor oral hygiene habits (*p* < 0.05).

**Conclusions:**

This study provides prevalence data on ST in a general population in France, which seems to remain high despite the existence of many therapies. It should alert professionals to a clinical manifestation that is becoming increasingly prevalent and that they will have to take into consideration to help reduce the discomfort arising from it.

## Background

Dentin hypersensitivity (DH) is a frequently recurring finding and a challenging condition to treat in clinical practice [1–3]. DH is characterized by an acute, transient pain from an area of exposed dentin, in response to stimuli that cannot be attributed to any other form of defect or disease. The stimulus is usually thermal, chemical, tactile, osmotic, or even related to evaporation (air jet) [2, 4].

Theories concerning the mechanisms of pain conduction inside the dentin are various and controversial. The hydrodynamic theory, developed in 1962 by Brännström, proposes the most commonly accepted explanation for this painful phenomenon [4, 5]. The activation of intra-pulp nerve fibres is thought to result from the displacement of the content of dentinal tubules following a mechanical, thermal or osmotic stimulus [5–7]. Two conditions need to be met for this to occur: the dentin has to be exposed and the dentin tubular system has to be open so as to allow the release of a neural response in the pulp through the fluid movement [7, 8]. The dentin tubule could be exposed by multifactorial interactions including erosion, abrasion, saliva, and biofilm/pellicle/plaque, all of which influence whether demineralization occurs or not [7]. Cervical enamel may be lost by a combination of erosion and abrasion, and recession of periodontal tissue may result in exposure of the root surface dentin [7]. Loss of periodontal tissue may also be the result of abrasion, as with tooth brushing, or periodontal treatment [7].

Symptoms of DH can sometimes resemble those of other painful oral diseases and diagnosis is mainly based on a search for predisposing factors, the identification of wear lesions and the elimination of other dental diseases such as caries or periodontal diseases [1, 8]. DH may affect the quality of life. It can influence what and how the subject eats and drinks, can hinder tooth brushing, and can even have severe emotional impacts and modify lifestyles [3, 9].

Different data syntheses on DH have shown prevalence ranging from 4 to 74% depending on the sample studied [10]. There may be several explanations for these variations. Some studies used self-administered questionnaires to ask subjects about sensitive teeth (ST) while others diagnosed DH after a professional clinical examination [10]. Persons declaring that they suffer from ST could be suffering not only from DH but also from other oral diseases such as caries or periodontal problems, which would explain the higher prevalence [10]. Nevertheless, even if studies are separated into two groups - self-questionnaires versus clinical records - the range of the estimate remains high (13–57% or 4–74% respectively) [10]. Other arguments have been put forward to explain these differences, such as sample characteristics (ethnic origin, study location, periodontal status, dental care regime, oral hygiene habits, socio-economic status) [10]. The clinical criteria used to define the presence or absence of DH could be based on two opposing approaches: a passive approach using the statement of the subject’s pain experience and an active approach applying different mechanical and thermal stimulations [11, 12]. Another complicating factor is the episodic nature of the condition, which may evoke or subdue the pain symptoms [3].

In France, published data on the prevalence of ST or DH are rare. In 1994, Murray and Roberts reported a prevalence of ST of 14% using a face-to-face questionnaire [13]. The objectives of the present work were to estimate ST prevalence and characteristics, and to explore the associated factors, in a general French population.

## Methods

### Description of the study

A multicentre cross-sectional questionnaire study was designed by the French Society for Dental Public Health (*Collège National des chirurgiens-dentistes universitaires en santé publique: CNCDUSP*). The study was conducted between November 2011 and March 2013 in six French cities (Bordeaux, Lille, Montpellier, Nice, Rennes and Strasbourg). The study protocol was drawn up in compliance with the STROBE guidelines for cross-sectional studies [14].

The coordinating team for the study (AB and JNV) read the available literature on the subject and wrote a first version of the questionnaire. An electronic questionnaire via the iSURVEY® application for iPad® (Apple Inc., California, USA) was used. The whole research team was invited to amend the questionnaire. Then, the amended questionnaire was tested by the 2 coordinators on a small number of people in order to identify difficulties in completing the questionnaire or understanding the meaning of each question or response. Any discrepancy was resolved by consensus discussion between the 2 coordinators, or the whole team if necessary. Once the questionnaire was finalized, a guide was edited to standardize data collection. The research team recruited two undergraduate dental students in each Faculty involved, to gather data. After training in both presentation of the study to eligible participants and filling in the questionnaire, the students conducted the questionnaire survey at the relevant places and times identified by their local supervisor. Students recorded the anonymous responses to questionnaires on an iPad®. On return to the Faculty, they transferred the data collected for their storage in a secure, web-based program.

In order to get a large sample of the general French population, adult passers-by were invited to participate in the study in public places (outside shopping centres, in city centre streets, on railway platforms). Subjects under 18 or under tutorship or curatorship, or who had severe difficulties in understanding spoken French, or who were edentulous were not included.

### Ethical considerations

For this type of street-survey study, the approval of an Ethics Committee was not mandatory in France during the period of construction and conduct of the study (Article L1121–1 of the *Code de la santé publique*, corresponding period). The data were collected anonymously. Each subject was first informed orally about the study (objective, method, interest and duration) and could ask any question. He or she was also informed of the possibility to stop and/or retry at any time during his/her participation in the study, and that the data were collected anonymously. Each person who agreed to participate gave his/her informed oral consent prior to participation in the study. Each subject was compensated for the time spent by the delivery of a brushing kit.

### Description of the questionnaire and the variables recorded

The questionnaire is presented in Additional file 1. All the questions were closed ended questions, except a few that had short answers. The primary outcome was the experience of ST during the previous 12 months (the question put to each participant was: “*have you experienced sensitive teeth over the last twelve months?*”). The following variables were collected to describe the sample characteristics: socio-demographic variables (gender, age, occupation and marital status), use of tobacco, diet habits (frequency of consumption of sodas, fruit juices and chewing gums, with / without sugar), oral hygiene habits (frequency of tooth brushing, type of equipment used), and access to oral care (time since last visit to the dentist, awareness of untreated oral problems). Reported frequent vomiting due to anorexia / bulimia, drugs, stress or pregnancy or periods of frequent gastro-oesophageal reflux were also recorded.

Other variables were collected only in subjects with ST. The level of pain was quantified using an electronic visual analogue scale. The participants were invited to express the intensity of their perceived pain by selecting a point on a horizontal line (100 mm) drawn between two ends, anchored by two verbal descriptors (at left, No pain and, at right, Maximum imaginable). Type of pain (acute, discomfort, pulsatile), occurrence characteristic (spontaneous or induced), perceived origin (cold, heat, sugar, air), duration (short or permanent or persistent), frequency (rare or occasional or frequent), oral habits affected by this pain (tooth brushing, diet, drinks) and lifestyle changes were also recorded. Variables were recorded to estimate the proportion of individuals who had mentioned this problem of ST to a health professional, and the benefits of the solutions proposed. Use of self-medication and its benefits were also evaluated.

### Sample size

Given the proportion of people with ST found in previous international epidemiological studies (*detailed in the Introduction section*), it was decided that 40% of people suffering from ST with a precision of ±2% was a reasonable basis for calculations. Alpha risk was fixed at 0.05. The minimum number of subjects to be included was calculated at 2305. So each city centre was requested to include at least 400 subjects, which gave a safety margin of 95 questionnaires.

### Statistical analysis

The database was accessed directly in Excel® format (Excel®11.0, Microsoft Corp, Washington, USA) once completed questionnaires had been downloaded from the iSURVEY® application for iPad® to the internet iSURVEY® site (iSURVEY, Wellington, New Zealand) via an internet connection. Statistical analyses were performed using Stata®9.0 software (Stata Corp LP, Texas, USA). No imputation on potential missing data was planned because the electronic questionnaire was designed to require an answer to each question, except those about ST characteristics.

The descriptive analysis of the sample was carried out using frequencies for categorical variables, and means and standard deviations for quantitative variables. Age was divided into 3 categories (18–34, 35–64, 65 years and over). It has already been shown that the association between age and oral health-related quality of life (OHRQoL) related to DH is not linear [15]. More precisely, the relationship between OHRQoL and age for patients with dentin hypersensitivity follows a specific pattern: oral health problems possibly increase slowly with age up to around 35–65 and then gradually fell again. By separating 3 periods of life, we sought to be able to identify a non-linear association, based on hypotheses from the literature. The frequency with which people reported ST during the previous 12 months and the 95% confidence interval (95% CI) were calculated. Answers from people with ST were further explored with the description of the type of pain, type of care offered by health professionals and use of self-medication. Differences between two groups were studied according to whether they reported having ST or not and according to other relevant variables identified with a significance level of 0.05 in the appropriate tests (Chi2 or Fisher test under conditions of application).

For the multivariate analysis, a logistic regression model was used. The dependent variable was the presence of ST. Independent variables with statistical significance at 20% or less in bivariate analyses, or any variable considered relevant, were included in the logistic regression analysis using a backward stepwise procedure. The results of the logistic analysis were presented with adjusted odds ratio (aOR) and 95% CI estimated for variables that remained in the final model.

## Results

The questionnaire was answered by 2413 persons, with well-balanced sample distributions among centres (Bordeaux: *n* = 399, Lille: *n* = 400, Montpellier: *n* = 417, Nice: *n* = 385, Rennes: *n* = 404, Strasbourg: *n* = 408). The total sample included 1255 women (52.0% of the sample) and the average age was 38.2 ± 15.3 years (median = 35.0, minimum = 18, maximum = 92).

The prevalence of ST during the previous 12 months reported in the sample was 42.2% [95% CI: 40.2–44.1%]. Prevalence of reported ST according to the investigation centre ranged from 31.2% in Rennes to 55.8% in Nice (with 34.8% in Montpellier, 42.3% in Lille, 43.6% in Bordeaux and 46.3% in Strasbourg). ST mean pain intensity assessed by a visual analogue scale was 3.8 ± 2.3 (median = 3.2, minimum = 0.1, maximum = 10, *n* = 1018). Pain characteristics, oral habits, quality of life affected by ST and solutions found to relieve pain are described in Table 1.

**Table 1:** Characteristics of the group who declared sensitive teeth (ST): pain characteristics, oral habits and quality of life affected by ST, solutions found (*n* = 1018)

	Number of subjects	Percentage (%)
**Total**	**1018**	**100**
**Type of pain** ^**a**^	1013	
Acute	326	32.2
Discomfort	577	57.0
Pulsatile	177	17.5
**Existence of a stimulus**	1014	
Spontaneous	304	30.0
Induced	710	70.0
**Origin of pain** ^**a**^	907	
Cold	746	82.3
Heat	187	20.6
Sugar	184	20.3
Air	253	27.9
**Duration of pain**	1013	
Short	761	75.1
Permanent	57	5.6
Persistent	195	19.3
**Frequency of pain**	1016	
Rare	332	32.7
Occasional	469	46.1
Frequent	215	21.2
**Oral habits affected by ST:**		
**Tooth brushing**	1015	
Yes	234	23.1
No	781	76.9
**Diet**	1016	
Yes	182	17.9
No	834	82.1
**Drinks**	1015	
Yes	275	27.1
No	740	72.9
**Lifestyle changes**	1013	
Yes	68	6.7
No	945	93.3
**ST discussed with a professional**	1014	
Yes	550	54.2
*With a medical doctor*	*28*	*5.1*
*With a dentist*	*513*	*93.3*
*Other*	*9*	*1.6*
No	464	45.8
**Benefit from the treatment proposed by the professional**	447	
Yes	390	87.2
No	57	12.8
**Use of self-medication**	979	
Yes	266	27.2
No	713	72.8
**Benefit from the use of self-medication**	256	
Yes	197	77.0
No	59	23.0

^a^ Non-exclusive answers to the question, i.e. multiple answers were possible

Results from the bivariate analyses according to the presence of ST are presented in Tables 2 and 3. Presence of reported ST was statistically associated with occupation (*p* = 0.01) and use of tobacco (*p* < 0.001) but not with gender, age group, marital status or a period of frequent vomiting (with respectively *p* = 0.14, *p* = 0.09, *p* = 0.69 and *p* = 0.32). The consumption of soft drinks and chewing gums were significantly associated with ST (with respectively *p* < 0.001 and *p* = 0.008). Regarding access to oral care, there was a significant association between ST and shorter time since last visit to the dentist, and between ST and awareness of untreated oral problems (with *p* = 0.005 and *p* < 0.001, respectively). Frequencies of tooth brushing and mouth washing, and type of toothbrush were also associated with ST (with, respectively, *p* = 0.02, *p* = 0.01 and p = 0.01).

**Table 2:** Socio-demographic and medical characteristics of the sample according to the two groups: with and without sensitive teeth (ST) (*n* = 2413)

	Without ST		With ST		***p*** ^***b***^
	Number of subjects	Percentage ^**a**^ (%)	Number of subjects	Percentage ^**a**^ (%)	
**Total**	**1395**	**100**	**1018**	**100**	
**Gender**					0.14
Female	708	50.7	547	53.7	
Male	687	49.3	471	46.3	
**Age group**					0.09
18–34	672	48.2	524	51.5	
35–64	623	44.6	440	43.2	
≥ 65	100	7.2	54	5.3	
**Occupation**					0.01*
Farmer	14	1.0	7	0.7	
Craftsperson, merchant, business leader	95	6.8	70	6.9	
Intermediate profession	131	9.4	78	7.6	
Employee	331	23.7	301	29.6	
Labourer	56	4.1	48	4.7	
Executive or highly intellectual activity	267	19.1	167	16.4	
Retired	156	11.2	87	8.5	
No activity	345	24.7	260	25.6	
**Marital status**					0.69
Married/ Cohabiting couple	834	59.8	590	58.0	
Single	418	30.0	320	31.4	
Divorced	105	7.5	84	8.2	
Widowed	38	2.7	24	2.4	
**Use of tobacco**					< 0.001*
Yes	435	31.2	410	40.3	
No	960	68.8	608	59.7	
**Period of frequent vomiting** ^**c**^					0.32
Yes	248	17.8	197	19.4	
No	1147	82.2	821	80.6	

^a^ Percentage of individuals in each class of the independent variables

^b^ Chi2 test according to the conditions of application or Fisher test comparing the presence of ST with each socio-demographic or medical variable

^c^ due to anorexia / bulimia, drugs, stress or pregnancy or a period of frequent gastro-oesophageal reflux

* *p* < 0.05

**Table 3:** Oral health related characteristics of the sample according to the two groups: with and without sensitive teeth (ST) (*n* = 2413)

	Without ST		With ST		***P*** ^***b***^
	Number of subjects	Percentage ^**a**^ (%)	Number of subjects	Percentage ^**a**^ (%)	
**Total**	**1395**	**100**	**1018**	**100**	
**Frequency of:**					
**Juice consumption**					0.37
At least once per day	455	32.6	359	35.3	
Occasionally to often	818	58.6	577	56.7	
Never	122	8.8	82	8.0	
**Soft drink consumption**					< 0.001*
At least once per day	214	15.3	200	19.7	
Occasionally to often	841	60.3	634	62.3	
Never	340	24.4	184	18.0	
**Chewing gums**					0.008*
At least once per day	292	20.9	252	24.8	
Occasionally to often	737	52.8	548	53.8	
Never	366	26.3	218	21.4	
**Access to oral care**					
**Time since last visit to the dentist**					0.005*
Less than 6 months	521	37.3	447	43.9	
About one year	477	34.2	317	31.1	
More than a year	397	28.5	254	25.0	
**Aware of untreated oral problems**					< 0.001*
Yes	337	24.2	426	41.9	
No	910	65.2	482	47.3	
Don’t know	148	10.6	110	10.8	
**Oral hygiene habits**					
**Tooth brushing frequency**					0.02*
At least once per day	1297	93.0	921	90.5	
Less than once per day	98	7.0	97	9.5	
**Mouth washing frequency**					0.01*
Regularly	152	10.9	134	13.2	
Sometimes	481	34.5	388	38.1	
Never	762	54.6	496	48.7	
**Type of toothbrush used** ^**c**^					0.01*
Manual Hard	116	8.3	106	10.4	
Don’t know	133	9.5	126	12.4	
Other	1146	82.2	786	77.2	

^a^ Percentage of individuals in each class of the independent variables

^b^ Chi2 test according to the conditions of application or Fisher test comparing the presence of ST with each oral health related characteristics variable

^c^ The variable “type of toothbrush” was constructed with the category manual hard brush versus other (manual medium or soft brush, electric brush), suspecting that the hard brush would tend to be associated with ST

* *p* < 0.05

In the final logistic regression model, gender, use of tobacco, frequency of soft drinks consumption, access to oral care (time since last visit to the dentist and awareness of untreated oral problems) and oral hygiene habits (mouth washing frequency and type of toothbrush used) showed significant statistical associations with ST (Table 4). ST was significantly more frequent among females and smokers (with respectively aOR = 1.27 [95% CI: 1.07–1.52] in women compared to men and aOR = 1.29 [95% CI: 1.08–1.54] in smokers compared to non-smokers). The higher the consumption frequency of soft drinks was, the greater was the risk of ST increase, with aOR = 1.45 [95% CI: 1.17–1.79] for those who consumed them occasionally to often and aOR = 1.72 [95% CI: 1.30–2.26] for those who consumed them at least once per day, compared to those who never consumed them. Time since last visit to the dentist was associated with ST when subjects had consulted less than 6 months previously compared to those who had consulted more than a year ago (aOR = 1.63 [95% CI: 1.30–2.03]). The use of a manual hard brush or an unknown type of brush were significantly associated with ST compared to other types of toothbrush (manual medium or manual soft or electric brush) with aOR = 1.42 [95% CI: 1.06–1.91] and aOR = 1.36 [95% CI: 1.04–1.80], respectively.

**Table 4:** Factors associated with sensitive teeth (ST): results from the final multivariate model (*n* = 2413) ^a^

	OR ^**b**^	95% CI	aOR ^**c**^	95% CI	***p***
**Gender**				1.07–1.52	0.006*
Female	1.13	0.96–1.33	1.27		
Male	1.00		1.00		
**Use of tobacco**					
Yes	1.49	1.26–1.76	1.29	1.08–1.54	0.005*
No	1.00		1.00		
**Consumption frequency of soft drinks**					
At least once per day	1.73	1.33–2.25	1.72	1.30–2.26	< 0.001*
Occasionally to often	1.39	1.13–1.71	1.45	1.17–1.79	0.001*
Never	1.00		1.00		
**Access to oral care**					
**Time since last visit to the dentist**					
Less than 6 months	1.34	1.10–1.64	1.63	1.30–2.03	< 0.001*
About one year	1.04	0.84–1.29	1.15	0.92–1.44	0.21
More than a year	1.00		1.00		
**Aware of untreated oral problems**					
No	0.71	0.54–0.93	0.67	0.50–0.89	0.006*
Yes	1.70	1.28–2.26	1.63	1.22–2.19	0.001*
Don’t know	1.00		1.00		
**Oral hygiene habits**					
**Mouth washing frequency**					
Regularly	1.35	1.04–1.75	1.35	1.03–1.80	0.02*
Sometimes	1.24	1.04–1.48	1.18	0.98–1.42	0.08
Never	1.00		1.00		
**Type of toothbrush use**					
Manual hard	1.33	1.01–1.76	1.42	1.06–1.91	0.01*
Don’t know	1.38	1.06–1.79	1.36	1.04–1.80	0.02*
Other	1.00		1.00		

^a^ The initial logistic regression model was constructed with ST as dependent variable and gender, age group, occupation, use of tobacco, frequency of soft drink consumption, frequency of juice consumption, frequency of gum chewing, time since last visit to the dentist, awareness of untreated oral problems, tooth brushing frequency, mouth washing frequency and type of toothbrush used as independent variables using a backward procedure. The final logistic regression model is presented in the table with ST as dependent variable and gender, use of tobacco, consumption frequency of soft drinks, time since last visit to the dentist, awareness of untreated oral problems, mouth washing frequency and type of toothbrush used as independent variables (likelihood ratio chi-square test (12 degrees of freedom) = 158.53, *p* < 0.001, pseudo R-squared = 0.048)

^b^ Non-adjusted Odds Ratio and 95% Confidence Interval (Woolf Method)

^c^ Adjusted Odds Ratio and 95% Confidence Interval

^*^ *p* < 0.05

## Discussion

The objectives of this study were to estimate the prevalence of ST in a general population in France and to explore associated factors. Nearly half of respondents in a large sample of French people had perceived ST during the previous year.

Many studies have explored DH around the world but data are rare in France. When this study began, only Murray and Roberts had published such data, which dated from 1994. They found a prevalence of 14% of ST evaluated using a face-to-face questionnaire in a French sample of 1000 randomly selected people [13]. They repeated the study in France during the next spring and found a lower prevalence, at 9% [13]. More recently, and concomitantly with our study, West et al. studied DH in 3187 adults in Europe, including 700 subjects from France, according to 3 methods (ST evaluation with a self-administered questionnaire, DH evaluated after cold air stimulation with the Schiff ordinal scale and the response to pain from the patient) [16]. They found a prevalence of 39.6% if DH was defined as reported DH on any tooth in response to cold air stimulation, 11.6% if DH was defined as patient’s response discreetly recorded by a clinical examiner to cold air stimulation at level 2 or 3 of the Schiff score, and 21.0% of declared ST using a self-administered questionnaire [16]. The last result is quite different from that obtained in this study. It may be explained by different selection criteria. In particular, West et al. recruited patients aged 15 to 35 from a routine dental examination in general dental practice and excluded patients if they had 5 teeth or less, were currently wearing orthodontic appliances or had cervical restorations [16]. In 2019, a meta-analysis by Favaro Zeola et al. indicated an estimate of DH of 11.5% [95% CI: 11.3%11.7%] for the fixed-effects model (which could be regarded as the “best estimate” in absence of heterogeneity) and 33.5% [95% CI: 30.2–36.7%] for the random-effects model (which could be regarded as the “average prevalence” from all studies) [17]. They also found that the type of participants included in the studies had a modifying effect, whereas the method of diagnosis, frequently suggested by the literature as an influential factor, did not explain the variability of the prevalence estimate [17].

About 18 and 27% of the sample reported having changed their eating habits for food and drinks, respectively. Drinks appeared to be the most often modified because of ST; it could be thought that subjects reduced, for example, very cold and/or acid drinks in order to limit discomfort/pain caused by this type of drinks or because they had discussed the impact of these elements with their dentist. Although a majority of people with DH noted little impact on their overall quality of life, some reported a significant impact. Bekes et al. also found that patients with DH reported considerably more impaired OHRQoL than subjects in the general population [9, 15]. It would have been interesting to further investigate behavioural changes due to ST and their links with OHRQoL perceived by subjects, using a new, specific and validated instrument: DHEQ (for quality of life measure for dentin hypersensitivity) or its short version, the 15-item DHEQ [18, 19]. It can be noted that, among people with ST, a little more than half had raised the issue with a healthcare professional and preferably a dentist. The treatment proposed for this problem was deemed beneficial by almost 90% of the subjects who used it. Fewer subjects with ST admitted having used self-medication (27%) and 77% were satisfied. The answers to these questions may be biased because those who presented the questionnaires were dental students, so subjects might have tended to under-report their self-medication and overestimate their recourse to the dentist. However, these results are encouraging from the point of view of the decision of people with ST to visit a dentist.

Studies usually evoke a higher prevalence of DH in women than in men, because women are usually more attentive to their overall health and particularly their oral health [11, 20]. In the present study, a higher risk of ST was also found in women, with aOR = 1.27 [95% CI: 1.07–1.52]. There is, nevertheless, conflicting evidence for the gender distribution of DH [21]. Splieth and Tachou argued that women tend to brush more intensively than men, and that they eat more healthy fruity food items, which are also erosive [21]. According to these authors, this combination of erosion and abrasion presents an ideal mixture of etiological risk factors for DH. Some studies report a higher prevalence of DH in smokers than in non-smokers, a prevalence that should be modulated according to the periodontal status [10, 16]. In this study, a significant association between ST and use of tobacco was also found (aOR = 1.29 [95% CI: 1.08–1.54]). The presence of ST is independently associated with a more recent visit to the dentist. The links between ST and access to oral care are difficult to interpret, since subjects with ST might have been to the dentist more recently because of ST. However, health is both a cause and consequence of the use of care. Time since last visit or awareness of untreated oral problems would, therefore, not be relevant to the analysis of the use of health care. It would have been appropriate to include the reason for the consultation.

It is reported in the literature that the prevalence of DH is currently increasing, probably because of changes in lifestyle and increasing risk factors [22]. DH is linked to enamel erosion, which is the main factor in tooth surface wear. It is characterized by a chemical dissolution of the tooth surface after acid attack of non-bacterial origin, extrinsic or intrinsic [23]. Several authors have shown that repeated acid action, such as a frequent consumption of fruit juices or sodas could ultimately lead to tooth erosion by extrinsic attack of the tooth surfaces and opening of dentinal tubules [24, 25]. The multiple analysis did not show a statistically significant association between the presence of ST and consumption of fruit juices. Conversely, a significant association was found between ST and consumption of soft drinks, and the higher the consumption frequency of soft drinks was, the greater was the risk of having ST compared to no consumption: aOR = 1.72 [95% CI: 1.30–2.26] for a daily consumption, aOR = 1.45 [95% CI: 1.17–1.79] if the consumption was occasional to often. Discussion in the literature points out that the daily consumption frequency, the time the soft drink remains in the mouth and the moment the acid drink is taken (during meals or between meals) have an effect on dental erosion [26–28]. Some general conditions inducing gastric reflux, such as stomach problems, or eating disorders like anorexia or bulimia, are intrinsic factors predisposing to dental erosion processes [29]. Finally, no association was found with a declared period of frequent vomiting and ST (*p* = 0.32) in this study, whereas West et al. found a significant association between DH reported on any tooth in response to cold air stimulation and reflux or repeated vomiting (*p* < 0.0001, [16]).

The exposure of the root level dentin follows, in turn, the occurrence of gingival recession, since the thin layer of supra-dentinal cement above this level is easily removed [30]. Thus, factors predisposing to DH at root level are the same as those that indirectly trigger gingival recessions. The links between ST and attitudes towards oral hygiene habits are complex and often discussed in the literature. Thus, improper tooth brushing not only in terms of technique but also in terms of duration or equipment used, or even periodontal treatment (such as scaling and root planing or surgery) could reveal DH by exposing the root dentin [1, 7]. The multivariate analysis showed that the use of a manual hard toothbrush for brushing was significantly associated with ST (aOR = 1.42 [95% CI: 1.06–1.91]), unlike other types of toothbrush. Conversely, a vicious circle between DH and changes in oral hygiene frequency, because of the pain, could result in the emergence of other oral problems and amplification of DH. In the present study, 23.1% of sufferers reported having changed their oral hygiene habits because of ST. A significant association was found between the frequency of tooth brushing and ST in the bivariate analysis, an effect that disappeared when other factors were taken into account in the multivariate analysis. Other determinants, such as tooth brushing technique or type of toothpaste, were not recorded, so it was not possible to verify if an inadequate technique or an excessively abrasive toothpaste could be associated with DH through progressive abrasion of the teeth [1, 31]. West et al. did not find an association between DH and brushing movement in the bivariate analysis [16] but these issues will probably need more exploration when new studies are designed.

A longstanding debate exists on the most appropriate term to use to describe DH. Though the correct term can be questioned, current usage and the accepted definition now suggest a consensus on the use of the term DH [2]. Nevertheless, the data collected in this work did not report DH but ST since a declarative questionnaire was used. Moreover, subjects reporting ST may have been suffering from oral diseases other than DH, such as untreated caries or periodontal diseases, which could not be confirmed by a clinical examination. This may have led to an overestimation of the true prevalence of DH. All data interpretation must necessarily take this limitation into consideration. Even when considering only DH, current methods employ non-gradable mechanical and cold metal/air/water/ice stimuli and use response scales that often rely on subject-investigator interaction that lacks validation [32]. A novel approach, dental quantitative sensory testing, based on controlled graded cold air stimuli, looks promising for obtaining reliable measurements of DH [32]. This could suggest a direction for further studies. Recent research has also focused on the role of illness beliefs and coping in the adjustment to DH [33], which could constitute a promising area of research for better understanding and effective management of people suffering from DH.

Another limit of this study was the sampling strategy. The study recruited the number of subjects indicated by the calculation of the number required. A geographically balanced sample was established in six geographic regions spread throughout France. Nevertheless, representativeness cannot be guaranteed since no random sampling was performed. In particular, the people recruited were passers-by, which could explain why the proportion of unemployed people was above the national average (25% versus about 10% [34]). This, in turn, could have had consequences on the estimation of the prevalence because of the complex links between oral health (in particular ST / DH) and socioeconomic status. Some authors have found a tendency for patients with DH to come from higher social groups [9].

## Conclusion

To conclude, this study provides prevalence data on ST and associated factors in multivariate analysis in a general population in France. It seems that prevalence of ST still remains high in the general French population despite the existence of many therapies. The information collected in this study, and more generally, about DH should alert new generations of professionals to a clinical manifestation that is becoming increasingly prevalent and that they will have to take into consideration to help reduce the discomfort arising from it.

## Supplementary information

**Additional file 1.**

## Data Availability

The supporting database generated during the current study is hosted by OSF.io and is available at this DOI: 10.17605/OSF.IO/MCJUH

## Abbreviations

DH: Dentin hypersensitivity
ST: Sensitive teeth
CNCDUSP: Collège National des Chirurgiens-Dentistes Universitaires en Santé Publique (French Society for Dental Public Health)
STROBE: Strengthening the reporting of observational studies in epidemiology
CI: Confidence interval
OR: Odds ratio
