# Supplementary material for: Prevalence of sensitive teeth and associated factors: a multicentre, cross-sectional questionnaire survey in France
Source: BMC Oral Health. 2020 Aug 26;20:234. doi: 10.1186/s12903-020-01216-1 (PMC7448347; doi:10.1186/s12903-020-01216-1)
Supplement: Supplementary file 1 — Additional file 1. [file 12903_2020_1216_MOESM1_ESM.docx]

**Sensitive Teeth Questionnaire**

*(Translation proposed by the authors for the publication. The original questionnaire was created for this study and administered in French, using the iSURVEY^®^ application for iPad^®^)*

1. What is today’s date?
2. What is your town of residence?
3. Where are you answering this questionnaire?
   1. Shopping mall
   2. Public space (street, park, square, etc.)
   3. University
   4. Other
4. What is the first letter of your family name?
5. What is the first letter of your first name?
6. What is your gender?
   1. Female
   2. Male
7. How old are you? (in years)
8. Do you currently have a professional activity?
   1. Yes
   2. No
9. What is your socio-professional category?
   1. Farmer
   2. Craftsperson, merchant, business leader
   3. Executive or highly intellectual activity
   4. Intermediate profession
   5. Employee
   6. Labourer
   7. Retired
   8. No professional activity
10. What is your marital status?
    1. Single
    2. Married/Cohabiting couple
    3. Divorced
    4. Widowed
11. Do you smoke?
    1. Yes
    2. No
12. If you answered “yes” to Q11, how many cigarettes do you usually smoke per day? (number)
13. Have you ever had a period of frequent vomiting due to anorexia / bulimia, drugs, stress or pregnancy or a period of frequent gastro-oesophageal reflux?
    1. Yes
    2. No
14. Do you drink/chew: (grid for the answers)

|  | Never | Occasionally | Once per week | Several times per week | Once per day | Several times per day |
| --- | --- | --- | --- | --- | --- | --- |
| Sodas |  |  |  |  |  |  |
| Juices |  |  |  |  |  |  |
| Gum |  |  |  |  |  |  |

1. Do you use: (grid for the answer)

|  | With sugar | Without sugar |
| --- | --- | --- |
| Sodas |  |  |
| Juices |  |  |
| Gums |  |  |

1. When did you last visit a dentist?
   1. Less than 6 months ago
   2. About a year ago
   3. More than a year ago
2. Do you think you have any untreated oral problems? (several answers possible)
   1. Problems of decayed teeth
   2. Gum problems
   3. Problems with dental protheses
   4. Problems of tooth alignment
   5. No
   6. I don’t know
   7. Yes, but I don’t know which problems
3. Do you have oral problems that are currently being treated? (several answers possible)
   1. Problems of decayed teeth
   2. Gum problems
   3. Problems with dental protheses dentures
   4. Problems of tooth alignment
   5. No
   6. I don’t know
   7. Yes, but I don’t know which problems
4. How often do you brush your teeth?
   1. Never
   2. Occasionally
   3. Once per week
   4. Several times per week
   5. Once per day
   6. More than once per day
5. Which type of toothbrush do you currently use?
   1. Manual toothbrush with soft bristles
   2. Manual toothbrush with medium bristles
   3. Manual toothbrush with hard bristles
   4. Electric toothbrush
   5. I don’t know
6. Do you use a mouth rinse?
   1. Never
   2. Occasionally
   3. Often
   4. Every day
7. Do you use interdental brushes?
   1. Never
   2. Occasionally
   3. Often
   4. Every day
8. Have you experienced sensitive teeth over the last twelve months?
   1. Yes
   2. No

***The participants who answered “no” to Q23 stopped their participation at this stage; only those who answered “yes” answered Q24 to Q45.***

1. Using the Visual Analogic Scale, can you quantify your maximum pain as a number between 0 (no pain) and 100 (maximum pain you can imagine)? (answer on a scale)
2. What type of pain is it? (several answers possible)
   1. Acute
   2. Discomfort
   3. Pulsatile
3. How does the pain occur?
   1. Spontaneously
   2. Induced
4. What causes the pain? (several answers possible)
   1. Cold
   2. Heat
   3. Sugar
   4. Air
5. What is the duration of the pain?
   1. Short
   2. Persistent
   3. Permanent
6. How often does the pain occur?
   1. Rarely
   2. Occasionally
   3. Frequently
7. Is your diet affected by this pain?
   1. Yes
   2. No
8. Are your drinks affected by this pain?
   1. Yes
   2. No
9. Are your oral hygiene habits affected by this pain?
   1. Yes
   2. No
10. Is your lifestyle affected by this pain?
    1. Yes
    2. No
11. Have you ever mentioned this problem of sensitive teeth to a health professional?
    1. Yes
    2. No
12. If you answered “yes” to Q34, to whom?
    1. A dentist
    2. A physician
    3. A nurse
    4. Someone else
13. What was the diagnosis? (free text answer)
14. Which solution did she/he propose? (free text answer)
15. Did you take the treatment proposed?
    1. Not applicable
    2. Yes
    3. No
16. Did you experience any benefit from this treatment?
    1. Not applicable
    2. Yes
    3. No
17. Have you used self-medication to relieve sensitive teeth?
    1. Yes
    2. No
18. How did you learn about the self-medication?
    1. Advertising
    2. Friends/family
    3. Other
    4. Not applicable
19. What kind of self-medication did you use? (free text answer)
20. Do you still use this self-medication?
    1. Yes
    2. No
    3. Not applicable
21. Do you feel any improvement with the self-medication?
    1. Yes
    2. No
    3. Not applicable
22. If you stopped the treatment, what were the reasons? (several answers possible)
    1. Cost
    2. Ineffective
    3. Disappearance of the symptoms
    4. Other
    5. Not applicable.
